# Multiple mosquito AMPs are needed to potentiate their antifungal effect against entomopathogenic fungi

**DOI:** 10.3389/fmicb.2022.1062383

**Published:** 2023-01-06

**Authors:** José L. Ramirez, Kylie J. Hampton, Alayna M. Rosales, Ephantus J. Muturi

**Affiliations:** ^1^Crop Bioprotection Research Unit, United States Department of Agriculture, Agricultural Research Service, National Center for Agricultural Utilization Research, Peoria, IL, United States; ^2^Biology Department, Bradley University, Peoria, IL, United States

**Keywords:** antifungal immunity, AMPs, fungal entomopathogen, mosquito immunity, *Aedes aegypti*, vector-pathogen interactions, antimicrobial effectors

## Abstract

Mosquito resistance to microbial infections, including fungal entomopathogens that are selected for mosquito control, depend on a range of antimicrobial effectors, among them antimicrobial peptides (AMPs). These short peptides, along the antimicrobial effector lysozyme, act by disrupting the microbial cell membrane or by interfering with microbial physiological processes. While the induction of AMPs and lysozyme during fungal entomopathogenic infections have been reported, their contribution to the mosquito antifungal response has not been evaluated. In this study, we assessed the induction of *Ae. aegypti* AMPs and lysozyme genes at two points of infection and against distinct entomopathogenic fungi. Our results indicate that fungal infection elicits the expression of cecropin, defensin, diptericin, holotricin, and lysozyme, but do not affect those of attacin or gambicin. We further evaluated the role of these antimicrobial effectors *via* RNAi-based depletion of select AMPs during challenges with two entomopathogenic fungi. Our results reveal that AMPs and lysozyme are critical to the antifungal response, acting in concert, rather than individually, to potentiate their antimicrobial effect against entomopathogenic fungi. This study further contributes to a better understanding of the mechanisms that confer resistance to entomopathogenic fungi in an important mosquito vector.

## 1. Introduction

Mosquito resistance to entomopathogenic microbes is dictated in part by potent antimicrobial effectors with ability to suppress a wide range of microbes (Frolet et al., 2006; Waterhouse et al., 2007; Yassine and Osta, 2010; Feng et al., 2020). Among these antimicrobial effectors we have the antimicrobial peptides (AMPs), molecules secreted by immune-competent tissues following a microbial infection or injury (Hillyer et al., 2004; Waterhouse et al., 2007; Mylonakis et al., 2016). Their production is under regulation of canonical innate immune signaling pathways (Imd and Toll pathway) and they are integral part of the humoral immune response designed to neutralize microbes that have breached the epithelial barrier (Lemaitre et al., 1997; Meister et al., 2005; Mylonakis et al., 2016). In that regard, the function of AMPs is thought to have evolved specifically to control persistent infections and as a complement to cellular and constitutive effectors that are launched first to control a microbial infection (Makarova et al., 2016).

Structurally, AMPs are a series of short cationic peptides that are classified according to their sequence and structures, such as the α-helical cecropins, the β-sheet cysteine-rich defensins, proline-rich drosocins, and glycine-rich attacins (Yi et al., 2014). The repertoire of mosquito AMPs include at least five families: defensins, cecropins, attacins, diptericin, and holotricin. The latest *Ae. aegypti* mosquito transcriptomic evaluation identified five cecropins, four defensins, five lysozymes, one attacin, one diptericin, one gambicin, and one holotricin (Hixson et al., 2022).

The defensins are peptides with an N-terminal loop and a α-helix linked to a β-sheet by two disulfide bonds, thus forming a loop-helix-beta-sheet structure (Yi et al., 2014). These cysteine-rich peptides range from 34 to 51 amino acids and have been found to be active against Gram-positive bacteria, Gram-negative bacteria, protozoan parasites, and fungi (Vale et al., 2014; Yi et al., 2014). The defensin peptide mode of action appear to be that of channel formation along the cytoplasmic membrane through interaction with the membrane phospholipids (Yi et al., 2014).

In comparison to this, the cecropins are α-helical linear peptide with a polarized N-terminal amphipathic helix with membrane-disrupting capabilities (Steiner et al., 1988). These peptides are about 4 kDa and constituted of 34–55 amino acids. Their pore-forming activity is fast, producing membrane lesions that allow cytoplasmic leakage and cell death (Lockey and Ourth, 1996; Brady et al., 2019). Cecropins have been shown to have activity against gram-positive and gram-negative bacteria, fungi, and protozoal parasites (Lockey and Ourth, 1996; Vale et al., 2014; Brady et al., 2019; Zdybicka-Barabas et al., 2019). In turn, the attacins have a mass of 23 kDa and have been shown to increase the permeability or to inhibit the synthesis of bacterial outer membrane proteins by binding their lipopolysaccharides (Vale et al., 2014; Yi et al., 2014; Buonocore et al., 2021). Like defensins and cecropins, attacins have been shown to be active against a range of microbes including gram-positive and gram-negative bacteria, fungi, and protozoal parasites (Yi et al., 2014; Buonocore et al., 2021).

Two other peptides, diptericin and holotricin, form part of the AMP repertoire in mosquitoes. Diptericins are glycine-rich peptides with about 8 kDa in mass and 82 residues (Keppi et al., 1989; Wu et al., 2018). This AMP is thought to have restricted activity toward a limited number of Gram-negative bacteria, acting on by disrupting the cytoplasmic membrane of a growing bacterium (Wu et al., 2018). Thus, diptericins are thought to have bacteriostatic properties through a potential interaction with cell membrane components involved in active amino acid uptake, which becomes bactericidal with time (Keppi et al., 1989). In turn, the less known holotricin is a 8 kDa glycine-rich peptide closely similar to defensins (Yi et al., 2014) and has been shown to be active mostly against Gram-negative bacteria (Lee et al., 1994; Imamura et al., 1999).

Another important antimicrobial effector, lysozyme, is often associated with both digestion and host response to microbial infection (Fiołka et al., 2005; Ursic Bedoya et al., 2005; Mohamed et al., 2016). Lysozymes are proteins whose mode of action is through the hydrolysis of β-(1,4) glycosidic bonds between *N*-acetylmuramic acid and *N*-acetylglucosamin of the peptidoglycan bacterial wall (Mohamed et al., 2016). Their antimicrobial activity is regarded to be stronger against Gram-positive bacteria than Gram-negative bacteria and have been shown to also have antifungal activity by hydrolyzing the β-(1,4) bonds of chito-oligosaccharides of fungal cell walls (Fiołka et al., 2005). In the mosquito *Ae. aegypti* some lysozymes have been found to be constitutively expressed and are further induced upon microbial challenge (Ursic Bedoya et al., 2005). Furthermore, lysozyme has been found to be acting synergistically with other AMPs to clear microbial infections (Chalk et al., 1994; Ursic Bedoya et al., 2005). For instance, the susceptibility of *Escherichia coli* to lysozymes become much more pronounced under the presence of attacins and cecropins (Engström et al., 1984; Chalk et al., 1994).

While many of these AMPs have been shown to be induced during bacterial, viral, fungal, and protozoal infections, their functional characterization in mosquitoes during fungal entomopathogenic infections has not been fully evaluated. In the microbe world, fungal entomopathogens are unique in that they are in a close arms race with their insect hosts, with both organisms constantly evolving their counterattack measures (Butt et al., 2016). The wide diversity of fungal entomopathogens discovered so far holds promise as sustainable and environmentally friendly alternatives to the use of synthetic insecticides for mosquito control. For instance, *Beauveria bassiana* and *Isaria javanica*, entomopathogenic fungi with extensive host ranges, have been found to be effective at colonizing and killing diverse mosquito species (Achonduh and Tondje, 2008; Kikankie et al., 2010; Blanford et al., 2011; Butt et al., 2016; Evans et al., 2018; Ramirez et al., 2018b).

In this study, we characterized the expression of mosquito antimicrobial peptides and lysozymes at 3 and 6 days post-infection with different strains of entomopathogenic fungi. We further evaluated the single contribution of these antimicrobial effectors to mosquito survival *via* RNA interference-based silencing. Additional assays confirmed fungal proliferation once the antimicrobial pressure exerted by AMPs was reduced *via* quadruple knockdown of select antimicrobial effectors. Our results reveal that AMPs are playing a crucial role in the antifungal response, acting in concert, rather than individually, to exert their antimicrobial effect on entomopathogenic fungi.

## 2. Materials and methods

### 2.1. Mosquito rearing

*Aedes aegypti* mosquitos (Rockefeller strain) were reared under standard rearing conditions at 28°C, 60–70% relative humidity, under a 12 h light/dark cycle. Adults were provided a 10% sucrose solution and larvae were maintained with a 2:1:1 rabbit food:liver powder:fish flakes diet. To produce eggs, adults were offered a blood meal using an artificial membrane feeding system (Chem Glass, New Jersey, NJ, USA) and bovine blood (HemoStat Laboratories, Inc., California, CA, USA). Three-to-five-day old female mosquitoes were used in all experimental assays.

### 2.2. Fungal strains and infection assays

Three entomopathogenic strains, *Beauveria bassiana* (MBC 076), *Beauveria brongniartii* (MBC 397) and *Isaria javanica* (MBC 524) were used to assess AMP gene expression during a fungal infection process. These were originally isolated from *Anticarsia gemmatalis* (Noctuidae, Lepidoptera); *Melolontha sp*. (Scarabaeidae, Coleoptera) and *Hypothenemus hampei* (Scolytidae, Coleoptera), respectively. Initial fungal cultures were started from 50 μl of fungal glycerol suspensions that are stored at −80°C. Fungi was cultured on Sabouraud dextrose agar and yeast extract (SDAY) medium and incubated at 26°C for 10–15 days. Conidial oil suspensions were prepared by scraping spores from the surface of cultures into 1 mL soybean oil, vigorously mixing by vortex for a minimum of 15 min and filtered through a cheese cloth to remove mycelia. Conidial concentrations were determined by direct counts using a Bright-line hemocytometer and compound light microscope and adjusted to a final concentration of 1 × 10^8^ conidia/mL. Mosquito bioassays were conducted as previously described (Ramirez et al., 2018a,b). Briefly, mosquitoes were cold-anesthetized and 50.6 nL of the adjusted conidial oil suspension was topically deposited to the ventral surface of the coxal region of mosquitoes, using a Nanoject III (Drummond Scientific, Philadelphia, PA, USA) micropipette. For determination of antimicrobial gene expression assays, 3–5 days old mosquitoes were used during infection bioassays. For gene silencing, 2–3 days old *Ae. aegypti* females were cold-anesthetized and injected with dsRNA, allowed to rest for 1 day and then infected with fungal spore suspensions.

The control group of mosquitoes were exposed to the same volume of soybean oil, devoid of fungal spores. For each experiment, fresh conidial suspensions were made from new batches of conidia. All treatment groups of infection assays were maintained under standard rearing conditions and supplemented with sterile 10% sucrose solution and a sterile cotton. Mosquito mortality was evaluated daily after infection and cadavers were removed from assay cages. Effects of silencing on infection survival were evaluated *via* four independent experiments, each conducted with different mosquitoes and fresh conidial suspensions, on separate dates. The significance of the survival curves was evaluated *via* Kaplan–Meier with Log-rank test (GraphPad 9.0.0, California, CA, USA). The LT50 and LT95 values were calculated by probit analysis in SAS 9.4, USA.

### 2.3. Analysis of antimicrobial effector gene expression

Gene expression analyses were conducted in whole mosquito bodies after infection. Antimicrobial peptide expression following entomopathogenic fungal infection was assessed in pools of 5 mosquitoes per treatment, collected at 3- and 6 days post-infection (dpi). These two time points were analyzed to determine the point of infection when multiple antimicrobial peptides were expressed. Pools of mosquitoes were homogenized in TRIzol (Invitrogen, Massachusetts, MA, USA) using a 3.2 mm macerating bead and TissueLyser II (Qiagen, Germany) and subsequently processed for RNA, following the manufacturer’s instructions. Quality and concentration of sample RNA was obtained *via* NanoDrop (ThermoFisher Scientific, Massachusetts, MA, USA). RNA was normalized across samples and cDNA synthesis was obtained using the QuantiNova Reverse Transcription Kit (Qiagen, Germany) using 1 μg of total RNA. One microliter of synthesized cDNA was combined with PowerUp SYBR Green Master Mix (Life Technologies, California, CA, USA) and gene specific primers (Supplementary Table 1), at a concentration of 300 nM, to run 10 uL qPCR reactions. The qPCR cycling conditions were those recommended for the master mix: holding at 50°C for 2 min and 95°C for 2 min, followed by 40 cycles of 95°C for 1 s and 60°C for 30 s. A melt curve stage at the end of the reaction was included. Each sample was analyzed in duplicates. The reproducibility of the results was measured *via* three independent experiments, each conducted with different mosquitoes and fresh conidial suspensions, on separate dates. The expression level of antimicrobial peptides was normalized using the ribosomal protein *Rps17* (AAEL004175) (Joubert et al., 2016; Dzaki et al., 2017) as a reference gene. The ribosomal protein Rps17 gene has been routinely used as a reference gene to assess *Ae. aegypti* transcript profiles (Joubert et al., 2016; Barletta et al., 2017; Ramirez et al., 2018b). Antimicrobial peptide gene expression levels were determined through SYBR Green chemistry in a QuantStudio 6 Flex Real-Time PCR System (Applied Biosystems, California, CA, USA) and the data was analyzed post-run using the ΔΔCt method (Livak and Schmittgen, 2001). The statistical significance of fold change values was determined on log2 transformed values *via* ANOVA with Dunnett’s post-test in Prism (GraphPad, California, CA, USA).

### 2.4. Double-stranded RNA synthesis and gene silencing

The RNA depletion of antimicrobial peptides was performed *via* RNAi as previously described (Ramirez et al., 2020). In short, the T7 promoter region was added to each target gene primer (Supplementary Table 2) in a reaction using cDNA templates from *Ae. aegypti*. dsRNA products from each AMP target gene were created using the HiScribe T7 Quick High Yield RNA Synthesis Kit (New England Biolabs, Inc., Massachusetts, MA, USA). The FLuc template DNA control used in these assays are those included in the kit. It is a linearized plasmid containing the firefly luciferase gene under a T7 promoter. Following dsRNA synthesis, the products were diluted with molecular-grade water to a concentration of 3 ug/uL solution. Two-to-three-day old *Ae. aegypti* females were cold-anesthetized and injected with 69 nL of the 3 μg/μL dsRNA solution into the thorax using a nano-injector (Nanoject III, Drummond Scientific, Philadelphia, PA, USA) to silence antimicrobial peptides. Silencing efficiency relative to dsFluc controls in un-infected mosquitoes was evaluated for each silenced gene *via* qRT-PCR at 3 days post-dsRNA injection (Figure 3 and Supplementary Table 2).

**FIGURE 1:**
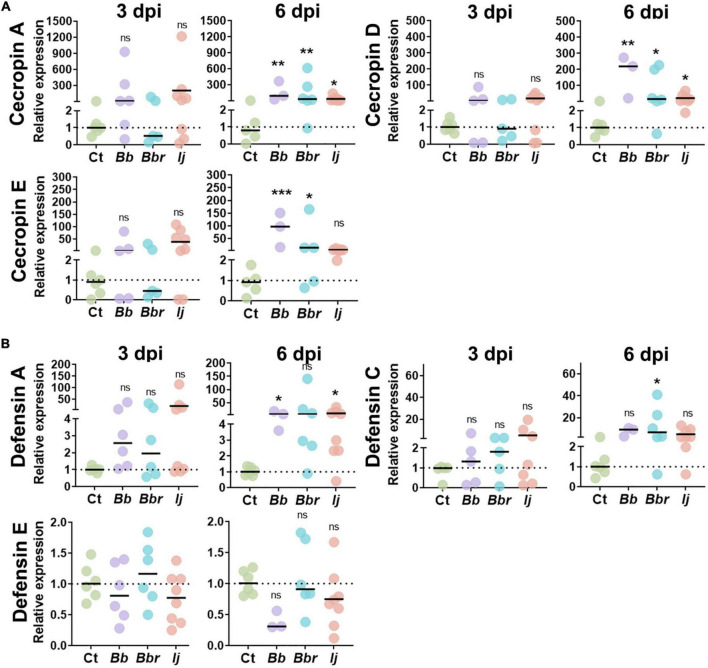
Induction of *Ae. aegypti* AMPs following infection with entomopathogenic fungi. Relative gene expression profiles of CecA, CecD, and CecE **(A)**, and defensin A, defensin C, and defensin E **(B)** in mosquitos infected with *B. bassiana, B. brongniartii*, and *I. javanica* at 3 and 6 dpi. The horizontal bar indicates the median level of expression from two independent experiments and each dot represents a pool of five mosquitoes. The statistical significance of relative gene expression was determined on log2-transformed values *via* ANOVA with Dunn’s multiple comparison test. *P < 0.05, **P < 0.01, and ***P < 0.001.

**FIGURE 2:**
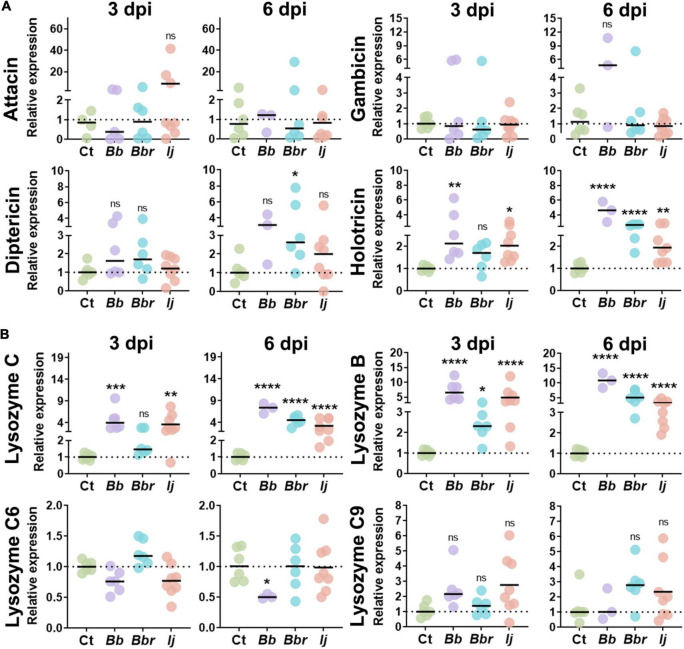
AMP and lysozyme induction in *Ae. aegypti* mosquitoes following infection with entomopathogenic fungi. Relative gene expression profiles of attacin, gambicin, diptericin, and holotricin **(A)**, and lysozyme C, lysozyme B, lysozyme C6, and lysozyme C9 **(B)** in mosquitos infected with *B. bassiana, B. brongniartii*, and *I. javanica* at 3 and 6 dpi. The horizontal bar indicates the median level of expression from two independent experiments and each dot represents a pool of five mosquitoes. The statistical significance of relative gene expression was determined on log2-transformed values *via* ANOVA with Dunn’s multiple comparison test. *P < 0.05, **P < 0.01, ***P < 0.001, and ****P < 0.0001.

**FIGURE 3:**
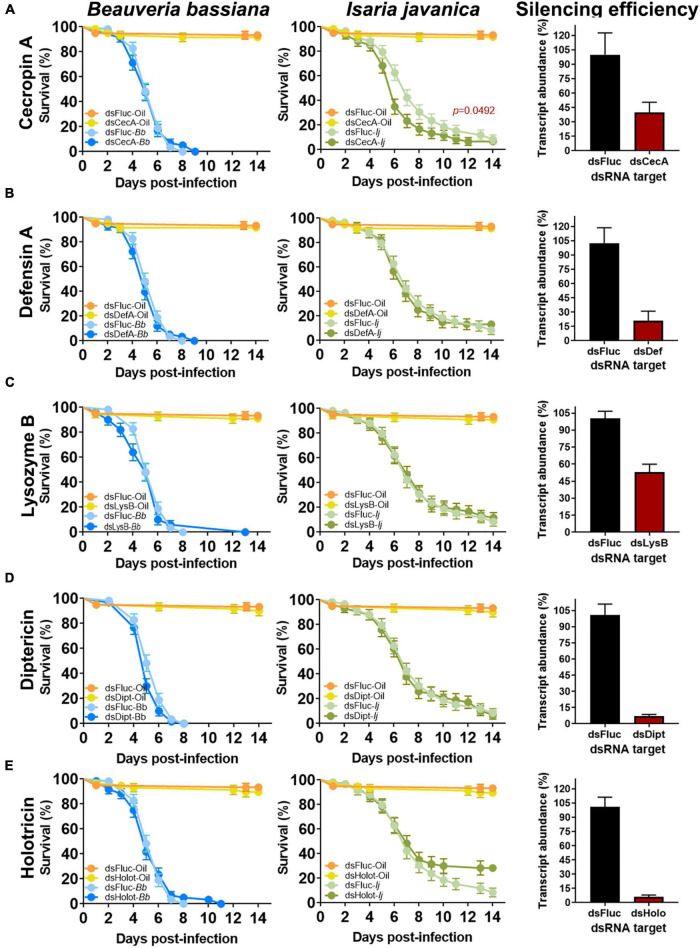
Effect of single AMP depletion on the survival of mosquitoes infected with entomopathogenic fungi. Silencing efficiency and survival curves of mosquitoes infected with *B. bassiana* and *I. javanica* post RNAi-knockdown of cecropin A **(A)**, defensin A **(B)**, lysozyme B **(C)**, diptericin **(D)**, and holotricin **(E)**. Survival curves represents at least four independent experiments and data was analyzed with Log-rank Test (GraphPad Prism 9). Error bars indicate the SEM.

**FIGURE 4:**
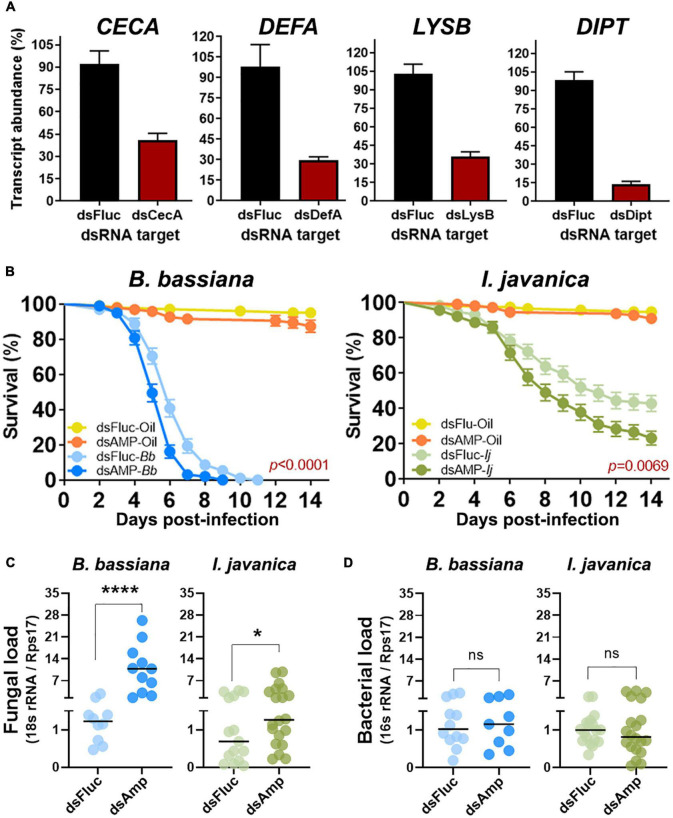
Quadruple AMP knockdown increases fungal proliferation and decreases mosquito resistance to entomopathogenic fungi. **(A)** Silencing efficiency at 5 days post-silencing for *CECA, DEFA, LYSB*, and *DIPT* during quadruple knockdown assays. **(B)** Survival curves of mosquitoes infected with either *B. bassiana* or *I. javanica* following quadruple AMP knockdown. **(C)** Fungal loads and **(D)** bacterial loads at 5 dpi in mosquitoes infected with either *B. bassiana* or *I. javanica* following quadruple AMP knockdown. Survival curves represents at least four independent experiments and data was analyzed with Log-rank Test (GraphPad Prism 9). Fungal loads were determined from two independent experiments and the statistical significance was determined on log_2_-transformed values *via T*-test. Error bars indicate the SEM. *P < 0.05, ****P < 0.0001, ns, not statistically significant.

Antimicrobial peptides were also silenced simultaneously to create a quadruple-knockdown of the AMPs that showed the most significant induction (cecropin, defensin, and lysozyme) or that had some impact on survival during single-knockdowns (diptericin, cecropin) to assess synergies in protection provided by the selected antimicrobial peptides. For simultaneous silencing, the dsRNA product for each AMP gene was adjusted to 3 μg/uL and then combined with other dsRNA products to create a final concentration of 12 μg/uL of pooled dsRNA. Fifty five nL of the pooled dsRNA treatment or dsFluc control (at the same concentration) was injected twice into the thorax of experimental mosquitoes using a nano-injector. Silencing efficiency relative to dsFluc controls was evaluated for each gene silenced in the quadruple-knockdown bioassays *via* qRT-PCR, using *Ae. aegypti* ribosomal protein *Rps17* as the reference gene for normalization, at 5 days post-infection in fungus-infected mosquitoes (6 days post RNAi injection). Gene silencing primers are reported in Supplementary Table 1. Silencing efficiencies are reported in Figure 4. Mosquitoes used in survival assays were challenged with two different strains of entomopathogenic fungi (*B. bassiana* or *I. javanica*) at 1-day post-dsRNA injection to assess the effect of AMP silencing on mosquito survival for 14 days.

### 2.5. Statistical analyses

Survival analyses and evaluation of gene expression post fungal infections were conducted using the software Prism 9 (GraphPad, California, CA, USA). Survival curves were evaluated *via* the Kaplan–Meier estimator with Log-rank test. Significance of gene expression among groups were evaluated on log2 transformed values *via* ANOVA with Dunnett’s post-test in Prism (GraphPad, California, CA, USA). Determination of LT_50_ and LT_95_ were obtained *via* probit analysis of mortality using SAS 9.4 statistical package. Statistical significance was assessed at *P* < 0.05, with the strength of the significance represented with asterisks (*P < 0.05; ^**^P < 0.01; ^***^P < 0.001) with the error bars indicating the standard error of the mean.

## 3. Results

### 3.1. Fungal infection induces antimicrobial peptide expression that varies with time of infection and fungal pathogen strain

To identify which antimicrobial effectors were elicited during fungal pathogenic infection, mosquitoes were infected with either *B. bassiana, B. brongniartii, I. javanica*, or soybean oil (control). We evaluated the expression of 15 different antimicrobial effectors *via* qPCR at two time points (3 and 6 dpi). These antimicrobial effectors belonged to the antimicrobial peptide groups cecropin, defensin, attacin, diptericin, gambicin, holotricin, and the antimicrobial protein lysozyme (Figures 1, 2).

Within the group cecropin, we evaluated the expression of cecropin A (*CECA*), cecropin D (*CECD*), and cecropin E (*CECE*) (Figure 1A). Although all three cecropins showed elevated levels of transcript abundance at 3 dpi during infections with *B. bassiana* and *I. javanica* (no change with *B. brongniartii* infections), they were only significantly induced at day 6 dpi. Overall mosquitoes infected with *B. bassiana* appeared to have significantly higher expression of *CECA, CECD*, and *CECE* relative to the control group. Mosquitoes infected with *I. javanica* induced *CECA* and *CECD* but did not induce *CECE*.

Our study evaluated three defensin AMPs, defensin A (*DEFA*), defensin C (*DEFC*), and defensin E (*DEFE*) (Figure 1B). Same as with cecropins, defensins did not show any significant level of induction to infection with any of the three entomopathogenic fungi tested at 3 dpi. At 6 days post-infection, the induction was elevated but their significance varied according to the fungal entomopathogen strain. For instance, mosquitoes infected with *B. bassiana* had a significant elicitation of only *DEFA*. In contrast, mosquitoes infected with *B. brongniartii* presented significant elicitation of one defensin (*DEFC*); while *I. javanica-*infected mosquitoes indicated induction of *DEFA*. Only defensin E did not show any level of significant transcript abundance change with any of the three fungal infection treatments.

We also evaluated the expression of four other antimicrobial peptides that have been identified in *Ae. aegypti*, attacin (*ATTA*), gambicin (*GAM*), diptericin (*DIPT*), and holotricin (*HOLO*). Gene expression analysis of *ATTA* and *GAM* indicated no significant elicitation of these two antimicrobial peptides with any of the fungal infection treatments or with time of infection. In contrast, we observed a significant induction of holotricin at 3 dpi in mosquitoes infected with *B. bassiana* and *I. javanica*, and no change in transcript expression in mosquitoes infected with *B. brongniartii*. These expression profile changed at 6 dpi where mosquitoes infected with any of the three fungal entomopathogens presented significant upregulation of *HOLO* expression. In particular, mosquitoes infected with *B. bassiana* and *B. brongniartii* presented strong *HOLO* upregulation relative to the control group. Diptericin expression presented more variation across fungal infection treatments, and while they showed elevated levels of infection, they were only significant in mosquitoes infected with *B. brongniartii* (Figure 2A).

Our panel of antimicrobial effectors also included lysozymes. We tested lysozyme C (*LYSC*), lysozyme B (*LYSB*), lysozyme C6 (*LYSC6*), and lysozyme C9 (*LYSC9*). Our analysis indicated a strong upregulation of *LYSC* in mosquitoes infected with *B. bassiana* or *I. javanica* at 3 dpi or 6 dpi. In comparison, mosquitoes infected with *B. brongniartii* only presented significant *LYSC* induction at 6 dpi. The gene expression profile for *LYSB* was similar to that of *LYSC*, except that it was also slightly upregulated at 3 dpi in mosquitoes infected with *B. brongniartii*. While no significant upregulation of *LYSC6* or *LYSC9* was observed with any of the fungal treatments, infection with *B. bassiana* led to a significant downregulation of *LYSC6* at 6 days post-infection.

### 3.2. Single AMP silencing fails to impact mosquito survival to diverse fungal pathogens

To evaluate the implication of AMPs on the antifungal defense, we conducted single silencing *via* RNAi knockdown of microbial effectors that showed significant levels of elicitation post-fungal infection. These set included *CECA, DEFA, LYSB, DIPT*, and *HOLO*. Gene silencing efficiency evaluated at 3 days post-silencing indicated a depletion of 58% on the expression of *CECA*, 80% for *DEFA*, 47% for *LYSB*, 93% for *DIPT* and 94% for *HOLO* (Figure 3).

No significant effect of single AMP depletion was observed in mosquitoes infected with *B. bassiana* in which *CECA* (Log-rank Mantel-Cox test, *X*^2^ = 0.004569, *P* = 0.9461), *DEFA* (Log-rank Mantel-Cox test, *X*^2^ = 0.7810, *P* = 0.3768), *LYSB* (Log-rank Mantel-Cox test, *X*^2^ = 0.4390, *P* = 0.5076), or *HOLO* (Log-rank Mantel-Cox test, *X*^2^ = 0.01011, *P* = 0.9199) were silenced (Figure 3). A marginal, albeit non-significant effect was observed in *B. bassiana*-infected mosquitoes that had *DIPT* depleted (Log-rank Mantel-Cox test, *X*^2^ = 3.553, *P* = 0.0594) (Figure 3D).

In comparison, silencing of *CECA* prior to infection with *I. javanica* led to a significant decrease in survival compared to ds*FLUC* control (Log-rank Mantel-Cox test, *X*^2^ = 3.869, *P* = 0.0492) (Figure 3A). Survival was not significantly affected in *I. javanica*-infected mosquitoes in which depletion of *DEFA* (Log-rank Mantel-Cox test, *X*^2^ = 0.0004207, *P* = 0.9836), *LYSB* (Log-rank Mantel-Cox test, *X*^2^ = 0.07703, *P* = 0.7814), *DIPT* (Log-rank Mantel-Cox test, *X*^2^ = 0.08119, *P* = 0.7757), or *HOLO* (Log-rank Mantel-Cox test, *X*^2^ = 3.202, *P* = 0.0736) were conducted (Figures 3B, C).

### 3.3. Potential synergistic cooperation of antimicrobial peptide-derived protection during fungal entomopathogenic infections

To explore whether a synergistic cooperation of antimicrobial effectors is needed to control fungal entomopathogens, we conducted a quadruple silencing targeting the most common antimicrobial effectors that had significant levels of gene expression post-fungal infection (*CECA, DEFA, LYSB*) and those that presented any effect of single AMP silencing on mosquito survival (*CECA*, significant effect; *DIPT*, marginal effect albeit not significant).

Quadruple silenced produced similar levels of gene silencing efficiency as those observed during single AMP silencing. Silencing efficiency with these group was conducted at 5 days post-infection to concurrently evaluate any changes in fungal load resulting from the antimicrobial effector depletion. We observed a silencing efficiency of 51% for *CECA*, 69% for *DEFA*, 67% for *LYSB*, and 85% for *DIPT* (Figure 4A).

Quadruple AMP depletion led to a significant decrease in survival in mosquitoes infected with either *B. bassiana* (Log-rank Mantel-Cox test, *X*^2^ = 18.88, *P* < 0.0001) or *I. javanica* (Log-rank Mantel-Cox test, *X*^2^ = 7.292, *P* = 0.0069) (Figure 4B). The LT50/LT95 also indicated to be lower for *B. bassiana*-infected mosquitoes compared to *dsFluc*-injected controls. The LT50 for *I. javanica-*infected mosquitoes was also observed to be significantly lower than the *dsFluc*-injected controls. The LT95 couldn’t be determined because mortality was lower than 95% at the end of the experiment (Table 1).

**TABLE 1:** Estimated LT_50_ and LT_95_ values from *B. bassiana* and *I. javanica*-infected mosquitoes in which *Fluc* (control) or four antimicrobial peptide transcripts were depleted *via* RNAi.

	Fungal strain			
	***B. bassiana* (MBC 076)**		***I. javanica* (ARSEF 5875)**	
**RNAi target**	**LT_50_ (95% CI)**	**LT_95_ (95% CI)**	**LT_50_ (95% CI)**	**LT_95_ (95% CI)**
*Fluc*	5.8 (5.6–5.9)	8.5 (8.2–8.9)	11.2 (10.8–11.7)	ND
*AMP*	5.0 (4.8–5.1)	6.9 (6.7–7.2)	9.1 (8.8–9.4)	ND

ND (not determined) denotes treatments where the LT values could not be estimated, due to low mortality.

To corroborate whether this mortality could be attributed to a significant increase in fungal proliferation due to the antimicrobial effector depletion, we evaluated fungal loads *via* RT-qPCR at 5 dpi. Our results indicates that quadruple AMP silencing results in the significant increase of fungal loads in *B. bassiana* and *I. javanica*-infected mosquitoes compared to *dsFluc* controls. The effect was more apparent in *B. bassiana*-infected mosquitoes (^****^p < 0.0001) than in *I. javanica*-infected mosquitoes (*p < 0.0425) (Figure 4C). Given that AMPs and lysozyme have antibacterial properties which could impact bacterial loads, and thus potentially affect mosquito mortality, we evaluated bacterial loads *via* RT-qPCR at 5 dpi in the same samples used to evaluate fungal loads. Our results show that there was no significant difference in the bacterial load of dsFluc controls and dsAMP mosquitoes under the context of two fungal infections (Figure 4D).

## 4. Discussion

Mosquito survival to microbial infections is determined in part by their ability to control invading pathogens *via* the action of multiple antimicrobial effectors, among them AMPs (Gillespie et al., 1997; Butt et al., 2016; Mylonakis et al., 2016; Zanchi et al., 2017). While some AMPs have been found to be induced in response to microbial infections, there is a scarcity of functional characterization of this group under the context of entomopathogenic fungal infections. In this study, we characterized the expression of main *Ae. aegypti* AMP families, along with the antimicrobial effector protein lysozyme, and evaluated their antifungal properties against two distinct fungal entomopathogens.

First, we assessed the induction of AMPs in mosquitoes infected with three different fungal entomopathogens at 3 days and 6 days post-infection. Overall, this analysis revealed that fungal entomopathogenic infections induce the expression of several representatives of the AMP families cecropin, defensin, diptericin, holotricin, and the antimicrobial effector lysozyme, but do not affect attacin or gambicin gene expression. Furthermore, the strength of the induction appears to be dictated by the fungal entomopathogenic strain and time post-infection, with significant AMP expression during the later stages of infection.

This pattern of temporal AMP elicitation with fungal entomopathogenic infection might reflect the temporal-associated induction of immune signaling pathways by entomopathogenic fungi, given that upstream immune signaling pathway components (i.e., pathogen recognition receptors) governing their expression were found to be elicited mostly at around 60–72 h post-infection (Ramirez et al., 2020). Thus, this induction profile might reflect the level of fungal-derived pathogen-associated molecular patterns present at a given time, along with its detection and processing by upstream pathogen recognition receptors. Alternatively, it might be the result of fungal-derived immune evasive mechanisms or fungal-derived manipulation of AMP expression given that other antifungal effectors such as *TEP22* have been observed to be induced at earlier time points of infection (Wang et al., 2015; Ramirez et al., 2018a).

In comparison to the expression of AMPs, two lysozymes (*LYSC* and *LYSB*) were strongly induced at earlier time points in all fungal pathogenic infections tested. This might indicate that lysozyme along with other antifungal effectors (i.e., *TEP22*) comprises the first line of antifungal defense, and that AMPs complement the antifungal repertoire later in the infection process. Previous studies have revealed that indeed certain lysozymes in the mosquito *Ae. aegypti* are constitutively expressed and are further induced upon microbial challenge (Ursic Bedoya et al., 2005). This would agree with what has been previously hypothesized, with cellular and constitutive effectors acting first following an infection, and with inducible AMPs being secreted later to neutralize surviving pathogens (Makarova et al., 2016).

Our results also hint to a level of anti-fungal specificity within each of the antimicrobial effectors. For instance, within the cecropins, while *CECA* and *CED* were induced with all three fungal entomopathogens tested, *CECE* was only significantly induced in infections with *B. bassiana* and *B. brongniartii*. Similar results were observed with defensins, where *DEFC* was significantly induced only during infections with *B. brongniartii*. This was also observed with the antimicrobial effector lysozyme, where *LYSC* and *LYSB* induction levels during infections with *B. brongniartii* lagged those observed with *B. bassiana* and *I. javanica* infections at the early stages of infection (3 dpi).

Next, to corroborate the antifungal potential of each of the AMP families, we conducted a RNAi-based depletion of select antimicrobial peptides that exhibited significant induction during fungal entomopathogenic infections. To our surprise, single antimicrobial effector depletion only had a significant effect on the survival of mosquitoes depleted of *CECA* and infected with *I. javanica*. This result is similar to what has been observed in *Drosophila*, in which single AMP depletion had marginal or no effect on an insect susceptibility to pathogens (Zanchi et al., 2017; Hanson et al., 2019). Furthermore, the strain-specific antifungal properties of cecropin have been described previously in *Drosophila*, in which the growth of *Metarhizium anisopliae* and the yeast *Saccharomyces cerevisiae* were inhibited while those of *B. bassiana* were unaffected by the action of cecropin (Ekengren and Hultmark, 1999). The fact that cecropin appears to be essential to control the growth of the less virulent *I. javanica* but not *B. bassiana*, might underscore a potential link between pathogenicity and resistance to AMPs that needs to be further studied.

It has been hypothesized that the existence of several AMPs corresponds to the need of multiple antimicrobial effectors necessary to successfully suppress a microbial pathogen (Zdybicka-Barabas et al., 2012; Hanson et al., 2019). Our data obtained through the quadruple-silencing of select AMPs (*CECA, DEFA, LYSB*, and *DIPT*) agrees with this hypothesis, with quadruple AMP depletion rendering mosquitoes more susceptible to infections by *B. bassiana* and *I. javanica* than single AMP knockdowns. This indicates that mosquito AMPs might be acting in concert to suppress entomopathogenic fungal growth. In fact, similar results have been observed in *Drosophila* (Hanson et al., 2019), in the beetle *Tenebrio molitor* (Zanchi et al., 2017) and the moth *Galleria mellonella* (Zdybicka-Barabas et al., 2012), where AMPs were found to act additively or synergistically to suppress bacterial growth. Whether mosquito AMPs are acting synergistically or additively to control fungal entomopathogens remains to be elucidated.

Our studies probing the fungal load in AMP-depleted mosquitoes further confirm the critical role AMPs play in suppressing fungal entomopathogen replication in mosquitoes. For instance, the increased susceptibility due to quadruple AMP depletion led to a 9.5-fold and 1.3-fold increase in the levels of *B. bassiana* and *I. javanica* fungal loads, respectively. The higher fungal loads observed in AMP-depleted mosquitoes infected with *B. bassiana* compared to *I. javanica*, might indicate that *B. bassiana* is much more susceptible to the action of antimicrobial effectors. Alternatively, it might reflect the fungal virulence of each of these fungi, given that samples were collected at 5 dpi, a time where *B. bassiana* is further along in their infection process compared to *I. javanica*. Our bacterial load assessment *via* qPCR further corroborates our findings, linking the increase in mortality of AMP-silenced mosquitoes to an intensification in fungal replication, and not to an increase in bacterial loads. Entomopathogenic fungal infections are known to increase the bacterial load of mosquitoes (Ramirez et al., 2018a), which could mask any effects of AMP silencing on the bacterial load of fungus-infected mosquitoes. Further studies using *in vitro* synthetized AMPs are needed to pinpoint the specific anti-fungal properties of these mosquito antimicrobial effectors. In summary, this work demonstrates the critical role that AMPs play in the antifungal defense. The AMP expression profile during infection with different entomopathogenic fungi indicates a time-dependent induction and appears to hint to a level of specificity. The fact that other antimicrobial effectors, such us *lysozyme* (this study) and *TEP22* (Wang et al., 2015; Ramirez et al., 2018a) have been found to be elicited at early times of fungal infection, might indicate that a somewhat delayed AMP expression is part of the mosquito antifungal strategy, with AMP recruitment as the second line of effector response to limit the spread of fungal infection. Furthermore, our study indicates that mosquito AMPs are working in unison to potentiate the anti-fungal response and suppress fungal entomopathogenic replication inside the mosquito hemocoel. This study further contributes to a better understanding of the mechanisms that confer resistance to entomopathogenic fungi in an important mosquito vector, and will allow us to improve fungal-based mosquito control strategies by integrating entomopathogenic fungal strains with distinct modes of action.

## Data availability statement

The original contributions presented in this study are included in this article/Supplementary material, further inquiries can be directed to the corresponding author.

## Author contributions

JR conceived and conducted the study, analyzed the data, and wrote the manuscript. KH and AR assisted with experimental bioassays, data analysis, and manuscript writing. EM assisted on the determination, analysis of time-dependent mortality data, and manuscript writing. All authors edited and approved the final manuscript.
